# Important Modification of the Apprenticeship System, by the Apothecaries' Company

**Published:** 1831-01-01

**Authors:** 


### LVIII.

### APOTHECARIES' COMPANY.

THE following queries, with their official answers, as published in the Medical Gazette of the present month, are so important, that we hasten to give them the utmost publicity. It will be seen that the system of apprenticeship has now received a final and fatal overthrow—an overthrow which, we hope, we have somewhat contributed to, by our unceasing opposition to that system. It will now be observed with pleasure, by every well-wisher to the prosperity of medical science, that the whole period of medical study is included in the term of apprenticeship. As for the salvo, or proviso of *permission* from the master of the pupil for attendance on lectures during the apprenticeship, it is a "vox et preterea nihil." No surgeon will dare to refuse this stipulation at the signing of the indenture—for this simple reason, that unless he make such a concession, he will have no pupil. We rejoice exceedingly in this regulation of the Society of Apothecaries. "*Perge pede quo cepisti.*"

"Query 1. Are the two years attendance on lectures and hospital practice meant to occupy two entire *successive* years; or is it meant that two winter sessions, commencing in October and ending in April, comprising four courses of lectures and hospital attendance during those two sessions, be sufficient to constitute the required time?

The Court of Examiners do not require that two *entire successive* years should be devoted to lectures and hospital practice; nor do they require four courses of lectures on any branch of medical science, nor any hospital attendance during the first year.

2. Will an apprentice be admitted to an examination who is permitted by his master to commence his studies in London at the end of the fourth year of his apprenticeship; or, in other words, would the last year, or half year of his apprenticeship, devoted to medical study in London, be considered as a completion of the five years' servitude?

An apprentice will be admitted to an examination who has been *permitted* by his master to attend *all* the required *lectures* and *hospital practice* during his apprenticeship; and the time thus spent will be considered as so much of his apprenticeship.

3. Is it required that an apprentice should be 21 years of age before he commences his course of study in London; or would it suffice if he be 21 years old at the time of his examination?

It is not required that an apprentice should be 21 years of age before he commences his course of study in London, but he must be of that age before he can be examined; and although an apprentice may, with the permission of his master, commence his attendance on lectures at any age, it is not adviseable that he should do so until he has completed his 19th year, unless he is an apprentice in London, in which case he may with advantage begin to attend lectures in the very first year of his apprenticeship.

4. Is a youth now in his medical apprenticeship considered as having commenced his study in medicine, and thereby exempt from the operation of the last or new regulations, in like manner as the pupil who commenced his attendance on lectures, &c. in London on the 1st of October, 1830?

The Court of Examiners have stated in the last regulations, that *all students* whose attendance on *lectures* shall commence on or after the 1st of Jan. 1831, must observe these regulations.

5. Would four winters, or four half years in regular succession, or at irregular periods, be computed as the two years' attendance in London, as required by the last or new regulations?

Four periods of six months each, either in regular succession or at irregular periods, will be computed as two years, provided the order of succession pointed out by the regulations has been observed by the student in his attendance on lectures and hospital practice.

6. In case of the illness of a student while prosecuting his medical studies in London, whereby a loss of time (say half a year) might occur, will he be required by that half year's loss of study to continue six months longer in London to complete the two years' attendance which the new regulations require; or would the half year's loss of study go unnoticed by the Court of Examiners?

When any case of this nature arises, the student should state all the circumstances attending it to the Court, who will give it such consideration as it may appear to merit.

7. Are the medical schools at Paris recognised by the Court of Examiners on the same grounds as those of Edinburgh, Dublin, &c.

They are.

